# Cæsarean Section on a Cow1Read before the annual meeting of the Iowa and Nebraska Veterinary Medical Association, November 20, 1900.

**Published:** 1901-07

**Authors:** J. G. Parslow

**Affiliations:** Shenandoah, Iowa


### CÆSAREAN SECTION ON A COW.[1]

By J. G. Parslow, V.S.,
SHENANDOAH, IOWA.

On June 11th last I was called to see a two-year-old heifer that had been trying for some time to calve. I found a medium-sized grade heifer in fairly good flesh and presenting a healthy appearance. She was lying in a shed, unable to rise, with very poor sanitary conditions surrounding her, and none better indoors to be had. I learned that she had been laboring some fifteen hours, the owner offering such assistance as he was able to by placing ropes on the two feet then presenting. I made an examination, and found them to be hind feet. The pelvic cavity was small, the calf large, plump, and an extra well-developed one. To make a normal presentation was beyond my power. To remove it through the natural channel was impossible without section, and section did not appear practical in this case, so I suggested removing by the side, though giving little hopes of favorable results. Consent was freely given, so I prepared to operate.

[1] Read before the annual meeting of the Iowa and Nebraska Veterinary Medical Association, November 20, 1900.

The patient was secured by stretching ; the right side sterilized. Chloroform was administered, with instructions to assistant how to follow the same. I then made an opening in the abdominal cavity, commencing a little below and about midway between the exterior angle of the ilium and the border of the last rib, running downward and slightly forward for sixteen inches. The uterus was then punctured, and the wall divided by force or torn across the superior surface. The fœtus and membrane were then removed, and the peritoneal coat of the uterus brought together by means of silk suture. The peritoneum closing the abdominal cavity received like stitches. The outside was closed by means of a heavy silk cord. Creolin dressings were used throughout.

The after-treatment consisted in sponging out the uterus on the fourth and fifth days, removing outside stitching on sixth day, and cleaning out and replacing same.

Internally sodium chloride in drinking water was all the treatment this patient received. She remained down six days, was then assisted to her feet each day for six days, since which time she has been taking care of herself, and is in good condition.

